# Evolving Dimensions of Bullying in Children

**DOI:** 10.3390/children11030305

**Published:** 2024-03-05

**Authors:** Muhammad Waseem

**Affiliations:** NYC Health + Hospitals, Lincoln Medical Center, Bronx, NY 10451, USA; muhammad.waseem@nychhc.org

Bullying remains a pervasive issue that affects many children worldwide, with devastating consequences that ripple through their lives and communities. The effects and consequences of bullying continue to evolve. There is a gap in the research that addresses the impact of bullying on the victim, the bully, and their families. This demands urgent attention and concerted efforts from parents, educators, policymakers, and society. This Special Issue focuses on school bullying during childhood and adolescence. The manuscripts referenced in this Special Issue address several aspects of bullying and its influence on children’s lives.

The first manuscript emphasizes the importance of peer relationships and their capacity to prevent bullying. Peer relationships are essential for a healthy environment. The presence of school bullying can have profound effects on children’s peer cooperation. Negative peer interaction can result in a hostile peer environment where students focus more on asserting dominance or avoiding victimization than cooperating. Cooperative learning demonstrated significant positive effects [1]. It also resulted in a positive change in peer relations and affective empathy [2]. Negative effects were reduced with increased support from teachers and parents. Effective support systems at school and home can mediate the impact of school bullying on peer cooperation.

The second manuscript assesses the association between bullying victimization and psychological distress. Although there is an association between bullying victimization and psychological distress, the underlying mechanism of this link is not clear. This manuscript assesses the impact of bullying on mental health, specifically depression, anxiety, and stress. There is a multidirectional relationship between bullying victimization and mental health issues. Bullying victimization not only leads to mental health issues, but children who already suffer from these issues (e.g., depression, anxiety, or stress) may also be more vulnerable to being targeted by bullies. Also, this co-occurrence of depression and anxiety leads to a worse prognosis [3]. Furthermore, it also explores whether cognitive emotion regulation (CER) strategies could be a potential mediator. This study supports the concept that while dysfunctional CER strategies may be mediated by the impact of bullying victimization on depression, anxiety, and stress, bullying victimization did not significantly influence functional CER strategies.

The third manuscript examines the role of physical activity in preventing bullying. The relationship between bullying and types of physical activity is important in understanding the dynamics of bullying and potential mitigating possibilities. Physical inactivity is a serious public health concern among children and is related to other psycho-social variables [4]. Physical activity may be a protective factor against bullying victimization. In general, physical activities that involve competition can help in developing a defense mechanism [5]. This could help develop and implement effective prevention and intervention strategies. The promotion of physical activity can be an important component in bullying prevention.

The fourth manuscript attempts to understand the contexts that exacerbate or attenuate the connection between bullying and children’s mental health. Marginalized and disadvantaged children are vulnerable to being victimized. This article enhances our understanding of bullying experiences among disadvantaged children. This study explores the interplay between school-level disadvantage, bullying involvement, and mental health. These results help develop interventions that target children in the most disadvantaged population.

The fifth manuscript recognizes the dimensions and contexts of bullying and understands which psychosocial dimensions the dynamics of bullying influence most. This manuscript explores the relationship between bullying perception and its psychosocial dimensions. It has been suggested that bullying impairs all health-related variables. Emotional and social dimensions have served mainly to mediate it.

The sixth manuscript evaluates intercultural differences based on age and gender. Bullying has significantly increased among children worldwide, across all cultures. This study shows that the behaviors associated with school bullying also differ according to the gender of those involved. Nonetheless, both boys and girls internalize social stereotypes. This study on bullying in Spain and Italy yielded significant practical implications for education and society. This information could help in developing consistent approaches among several other countries. Addressing school bullying through a gender-oriented lens and trying to accommodate their distinct characteristics when developing strategies can be very important.

The seventh manuscript explores the association between witnessing bullying and internalizing its symptoms and how they could develop negative consequences. For example, depressive symptoms were evident among males and females who witnessed school bullying. Gender may be a moderator in the relationships between internalizing symptoms, witnessing school bullying, and defending associated behavior. Witnessing school bullying could help to predict depressive symptoms. Among bystanders, gender differences were noted in internalizing symptoms, particularly concerning the type of social anxiety. Specifically, among females, Social Avoidance and Distress were positively related to witnessing school bullying, and in males, defending behavior was positively related to the Fear of Negative Evaluation.

The eighth manuscript assesses the influence of bullying on positive emotions (PEs). Students’ positive emotional experiences significantly impact their academic performance (AP). PEs serve as a powerful mediator in determining their AP. Also, Internal Controllable Attributions (ICAs) are correlated with positive emotions and academic performance. This study showed a close relationship between all three variables: AP, PE, and ICA.

The ninth manuscript looks at the impact of the visible nature of diseases and their negative effects. Several medical conditions can make children vulnerable to bullying, particularly diseases with esthetic and potentially disfiguring effects, such as Neurofibromatosis. Assessing the consequences of bullying behaviors in terms of psychological symptoms (i.e., depression and anxiety), Quality of Life (QOL), and self-esteem is warranted. These children are more likely to be victimized, and diseases such as these may also reduce their psychosocial QOL. Their interpersonal relations may also be affected by such stigmatization.

The tenth manuscript explains how empowering children reduces bullying. Empowering children and giving them tools against bullying are essential for their psychological well-being. Teaching children effective interventions and prevention strategies is critical.

In conclusion, this document addresses the mental health implications of bullying. There continues to be an urgent need for the further exploration of variables and mechanisms related to bullying behavior. There are still many variables and mechanisms that need to be explored. 
